# Characterization of cadmium-responsive MicroRNAs and their target genes in maize (*Zea mays*) roots

**DOI:** 10.1186/s12867-019-0131-1

**Published:** 2019-05-02

**Authors:** Jian Gao, Mao Luo, Hua Peng, Fabo Chen, Wenbo Li

**Affiliations:** 1 Centre for Green Development and Collaborative Innovation in Wuling Mountain Region, Yangtze Normal University, Fuling, Chongqing, 408100 China; 2 Department of Life Sciences and Technology, Yangtze Normal University, Fuling, Chongqing, 408100 China; 3 Key Laboratory of Medical Electrophysiology of Ministry of Education, Drug Discovery Research Center, Southwest Medical University, Luzhou, 646000 Sichuan China; 4 Laboratory for Cardiovascular Pharmacology, Department of Pharmacology, School of Pharmacy, Southwest Medical University, Luzhou, 646000 Sichuan China; 5 Sichuan Tourism College, Chengdu, 610100 Sichuan China

**Keywords:** miRNAs, Cadmium stress, Maize (*Zea mays*), Target gene

## Abstract

**Background:**

Current research has shown that microRNAs (miRNAs) play vital roles in plant response to stress caused by heavy metals such as aluminum, arsenic, cadmium (Cd), and mercury. Cd has become one of the most hazardous pollutants in the environment. Maize can be a potential model to study phytoremediation of Cd-contaminated soil owing to its large biomass production. However, little is known about miRNAs as a response to Cd stress in maize.

**Results:**

To investigate the role of miRNAs in response to Cd stress, roots of seedlings of the inbred maize lines B73 and Mo17 were collected and treated with 200 mg/L CdCl_2_·2.5 H_2_O over different exposure times. Enzyme activities of superoxide dismutase and peroxidase were measured to confirm Cd stress. The expression of six candidate miRNAs and their targets were validated using quantitative real-time PCR (qRT-PCR) technology. In addition, the expression of *Zma*-*miR171b* was assessed using in situ hybridization.

**Conclusions:**

Our results showed that miRNAs and their respective target genes were differentially expressed in maize seedling roots exposed to Cd stress. This research produced new insights into the molecular mechanism of miRNAs responsive to Cd stress in plants and sheds light on the latent roles of miRNAs in plants exposed to heavy metal stresses.

**Electronic supplementary material:**

The online version of this article (10.1186/s12867-019-0131-1) contains supplementary material, which is available to authorized users.

## Background

Contamination of soils with heavy metals has become a prevalent topic in ecology owing to their widespread release from industry, agriculture, and other human activities [1]. Cadmium (Cd) adversely affects various physiological and biochemical processes such as nitrogen metabolism, photosynthesis, mineral utilization, carbohydrate metabolism, and water uptake, thereby inhibiting plant growth and development [2, 3]. Cd pollution has substantially increased over the least four decades worldwide, which is a matter of concern for human health [4]. Therefore, research on the molecular mechanisms underlying Cd pollution in plants is urgently required.

Recently, several studies investigated the molecular mechanisms association with Cd tolerance and toxicity in plants [2, 3]. Exceptional microRNAs (miRNA) expression was identified in tissues and cells of stressed plants, and the involvement of miRNAs in Cd-stress responses was suggested [5–7]. In rapeseed under Cd stress, the miRNA *bna*-*miR393* was specifically expressed in leaves, *bna*-*miR156a* and *bna*-*miR167a/c* were expressed in roots and leaves, respectively, and *bna*-*miR164b* and *bna*-*miR394a/b/c* were expressed in all tissues [5]. Similarly, differential expression of numerous non-conserved and conserved miRNAs was reported to be induced by Cd stress in *Brassica napus* roots [5]. In the same study, 84 conserved and non-conserved miRNAs families with differential expression were identified in shoots and roots under Cd stress [5]. In rice, 19 Cd-responsive miRNAs and their targets including transcription factors and stress-related proteins were identified in seedlings using a microarray assay [6]. In addition, to assess Cd-responsive miRNAs and their associated genes in radish (*Raphanus sativus*), small-RNA libraries were constructed from plants under Cd stress and from controls. A total of 23 miRNA families with notable differential expression (15 previously known and 8 novel miRNA families) was identified in Cd-stressed plants, and their targets were then predicted using a degradome analysis [7]. Numerous potential heavy Cd-responsive miRNA candidates were identified in rice, *B. napus*, radish, and many other plants [5–7]. However, information on the molecular mechanism of Cd stress resistance in maize is limited. Therefore, we performed a characterization of Cd stress-mediated miRNAs and their target genes in roots of B73 and Mo17 maize under Cd stress and in negative controls. We identified target genes associated with Cd-regulated candidate miRNAs in maize seedling roots. The candidate miRNAs that respond to Cd stress will be useful for further improving Cd tolerance in maize.

## Results

### Expression pattern of conserved miRNAs in plant under different heavy metal stress

**Fig. 1:**
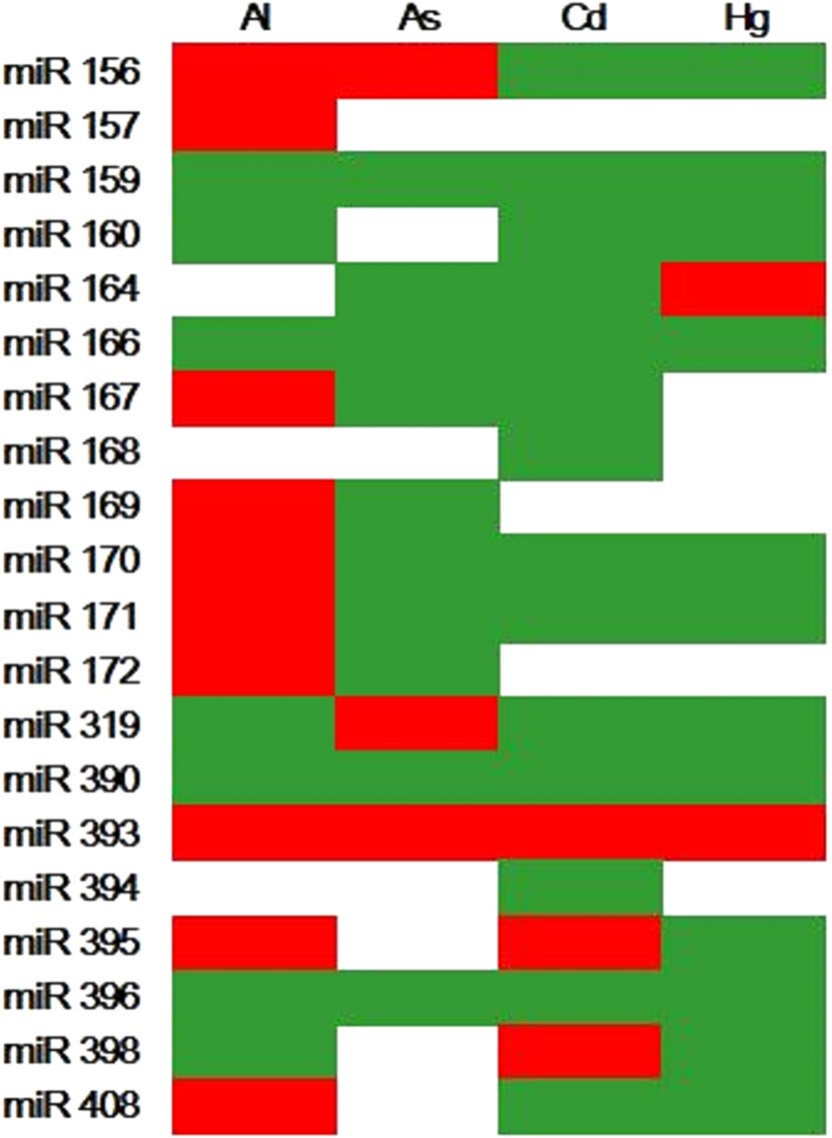
Altered expression of miRNAs in plants in response to heavy metal stress. Green indicates down-regulation, and red indicates up-regulation. Quantitative data of miRNA abundance in diverse plant species were collected; green indicates down-regulated miRNAs, red indicates up-regulated miRNAs

Recent studies showed that miRNAs play vital roles in regulating the expression of stress response genes following stress caused by various heavy metals. In this study, we assessed the expression patterns of miRNAs in plant exposed to different heavy metals, including aluminum (Al), arsenic (As), cadmium (Cd), and mercury (Hg) [6, 8–11]. Significant differential expression of miRNAs due to heavy metal stress was observed, such as down-regulation of common miRNAs (*miR159*, *miR160*, *miR166*, *miR319*, and *miR396*) and up-regulation of miRNA *miR393*. Other miRNAs also showed differential expression in response to heavy metal stress: *miR156*, *miR159*, *miR160*, *miR166*, *miR167*, *miR171*, *miR319*, *miR390*, *miR394*, *miR396*, and *miR408* were down-regulated, and *miR393* was up-regulated under Cd stress (Fig. 1). A conservation analysis of those miRNAs showed that *miR156/miR157*, *miR159/319*, *miR166*, and *miR170/miR171* were higher conserved in all organisms, followed by *miR160, miR396*, *miR167*, and *miR390*. The miRNAs *miR398* and *miR394* were less conserved in all organisms (Additional file 1: Table S4). Based on the results of significant differential expression and conservation analyses of these miRNAs under Cd stress, *miR156*, *miR166*, *miR167*, *miR171*, and *miR393* were selected as candidates for differentially expressed miRNAs responsive to Cd stress. In addition, we analyzed the expression profiles of these miRNAs and their target genes in the inbred maize lines B73 and Mo17 using qRT-PCR.

### Alterations in the development of maize leaves and root system in response to Cd stress

To confirm the appropriate treatment time for validation of candidate Cd-regulated miRNAs, we analyzed the enzymatic activities of superoxide dismutase (SOD) and peroxidase (POD) in Cd-treated maize roots and leaves after treatment times of 6–96 h. We found that SOD activity increased after 12, 24, and 48 h of the Cd-treatment, and particularly so after 12 h, in both Mo17 and B73. POD activity showed the opposite pattern in both phenotypes. After 48 h, SOD and POD activity gradually decreased to a constant level. SOD and POD activity was higher in roots than in leaves, in both phenotypes (Additional file 2: Figure S1). These results suggest that certain Cd-tolerant mechanisms were activated to maintain the normal physiological and metabolic activities shortly after the Cd treatment. Subsequently, RNA was isolated from the B73 and Mo17 treatments and control groups.

### Cd-regulated miRNA expression levels

**Fig. 2:**
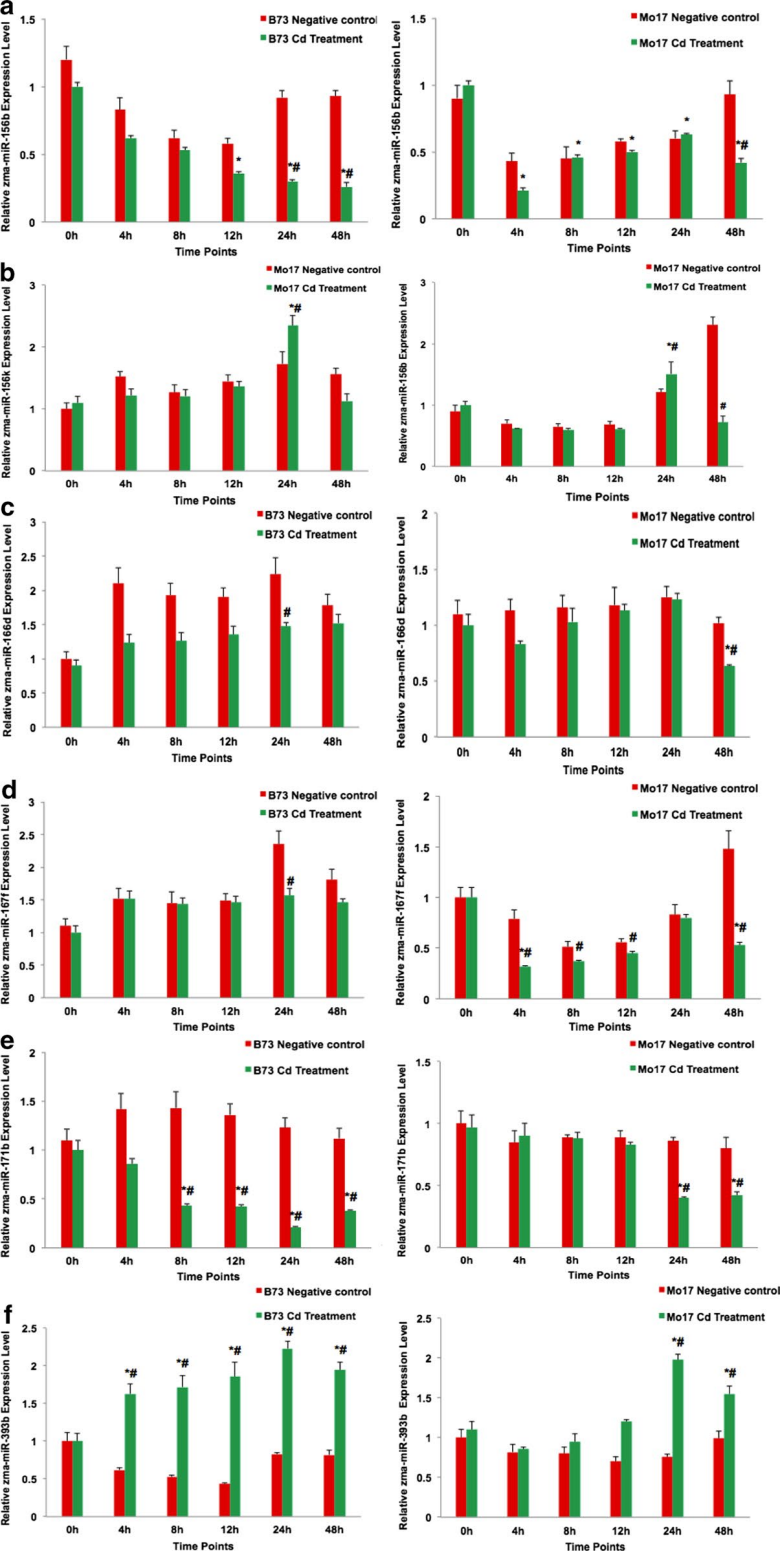
Validation of candidate microRNAs related to Cd stress using qRT-PCR. Left: B73, right; Mo17. Red and green vertical bars indicate the negative control (CK) and the Cd treatment, respectively. The letters A to F indicate the expression levels of *Zma*-*miR156b*, *Zma*-*miR156k*, *Zma*-*miR166d*, *Zma*-*miR167f*, *Zma*-*miR171b*, and *Zma*-*miR393b*. Shown are the mean ± SEM. *p < 0.05 Cd exposure time vs. 0 h; ^#^p < 0.05 treatment vs. negative control

To assess the functions of these candidate miRNAs selected according to altered expression levels of conserved miRNAs in plants under Cd stress, we analyzed their expression profiles using qRT-PCR. *Zma*-*miR156b*, *Zma*-*miR156k*, *Zma*-*miR166d*, *Zma*-*miR167f*, *Zma*-*miR171b*, and *Zma*-*miR393b* were expressed in maize roots of both maize lines (Fig. 2). *Zma*-*miR156b* showed a trend of down-regulation in roots after the Cd treatment in B73, compared with the control, whereas no significant changes were observed in Mo17 (Fig. 2a). *Zma*-*miR156k* showed no significant alterations in B73 and Mo17 (Fig. 2b). *Zma*-*miR166d* was more down-regulated in B73 than in Mo17 roots under Cd stress (Fig. 2c), and *Zma*-*miR167f* was more down-regulated in Mo17 than in B73 (Fig. 2d). *Zma*-*miR171b* was down-regulated in both maize lines (Fig. 2e), in contrast to *miR393b* (Fig. 2f).

### qRT-PCR analysis of target genes related to candidate microRNAs in response to Cd stress

**Fig. 3:**
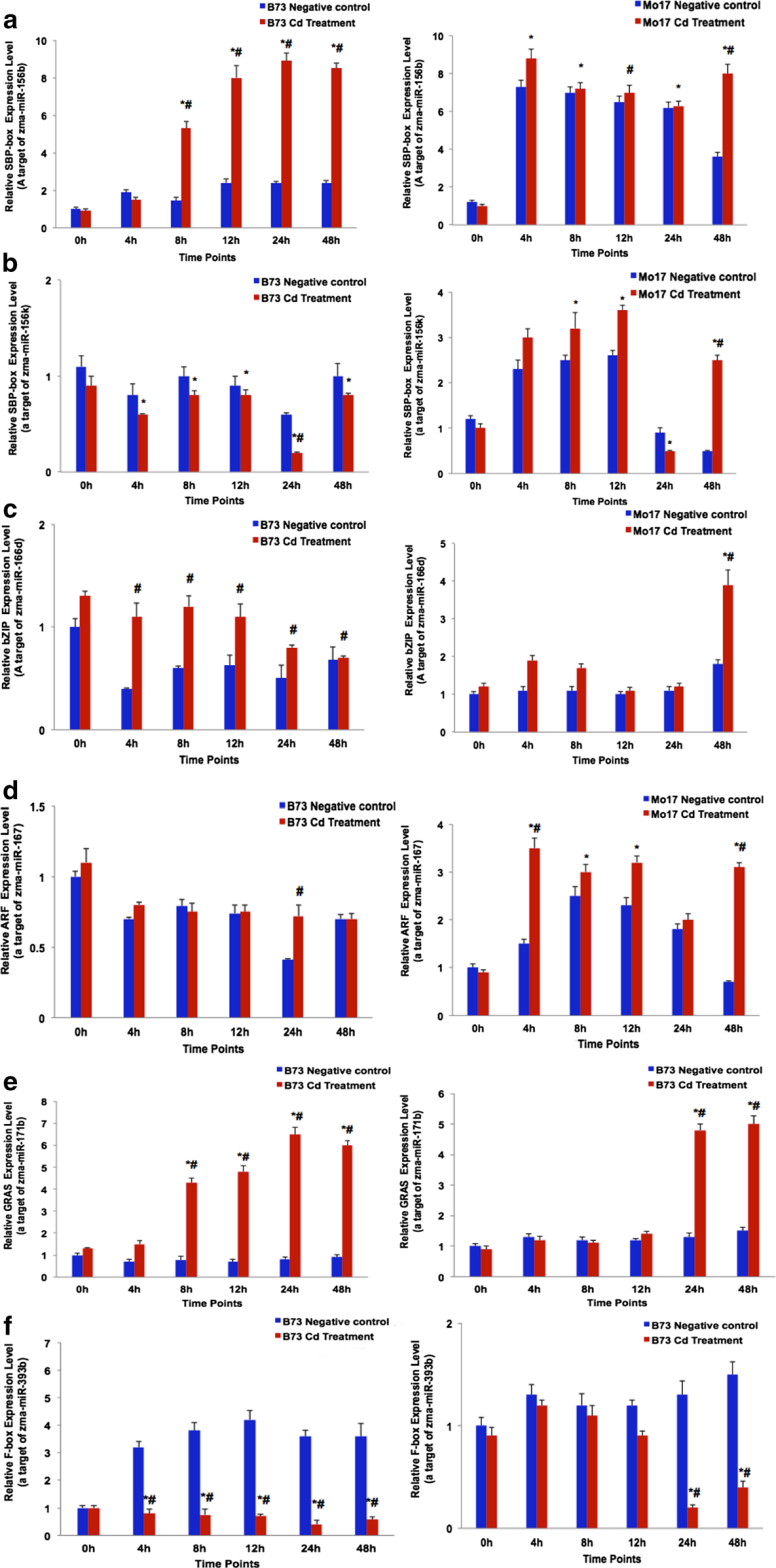
Validation of target genes of candidate microRNAs related to Cd stress using qRT-PCR technology. Negative controls (CK) sample and Cd-treated plants. The color nodes indicate the expression of fold change. Letters from A to F indicate expression levels of SBP-box, SBP-box, bZIP, ARF, GRAS, and F-box-like protein, respectively. Error bars indicate the standard error calculated from three biological replicates. Shown are the mean ± SEM. *p < 0.05 treatment time vs. 0 h; ^#^p < 0.05 treatment vs. negative control

The results suggested that the activities of miRNA-mRNA complexes could be altered by stress. To identify target genes of candidate miRNAs regulated by Cd stress, the target genes were predicted using WMD3 and PsRNA target website using the MGC database (ZmB73_v5b, Table. S5). Most of the target genes association with the candidate miRNAs belonged to transcription factor families, e.g., *Zma*-*miRNA156* targeting the SBP transcription factor family, *Zma*-*miR166* acting on the basic-leucine zipper (*bZIP*) transcription factors, *Zma*-*miR167* regulating auxin response factors (*ARFs*), GRAS transcription factors were predicted targets of *Zma*-*miR171*, and F-box proteins were predicted targets of *Zma*-*miR393*. The relative expression level of target genes seemed negatively correlated with that of the respective miRNAs (Fig. 3). The target genes of *Zma*-*miR156b* (*SBP*-*box*), *Zma*-*miR166d* (*bZIP*), and *Zma*-*miR171b* (*GRAS*) were significantly up-regulated during the dynamic development of roots in B73, compared to in Mo17 (Fig. 3a, c, e). In contrast, the target gene of *Zma*-*miR393b* (F-box-like protein) was observed to be down-regulated in B73 than in Mo17 samples (Fig. 3f). However, the target genes of *Zma*-*miR156k* (*SBP*-*box*) and *Zma*-*miR167f* (*ARF*) showed no significant up-regulation in either maize line (Fig. 3b, d). In conclusion, candidate miRNAs such as *Zma*-*miR156b*, *Zma*-*miR166d*, *Zma*-*miR171b*, and *Zma*-*miR393b* may negatively regulate their respective target genes in maize under Cd stress, which likely involves transcriptional and post-transcriptional regulatory networks.

### Expression of *Zma*-*miR171b* identified by in situ hybridization

**Fig. 4:**
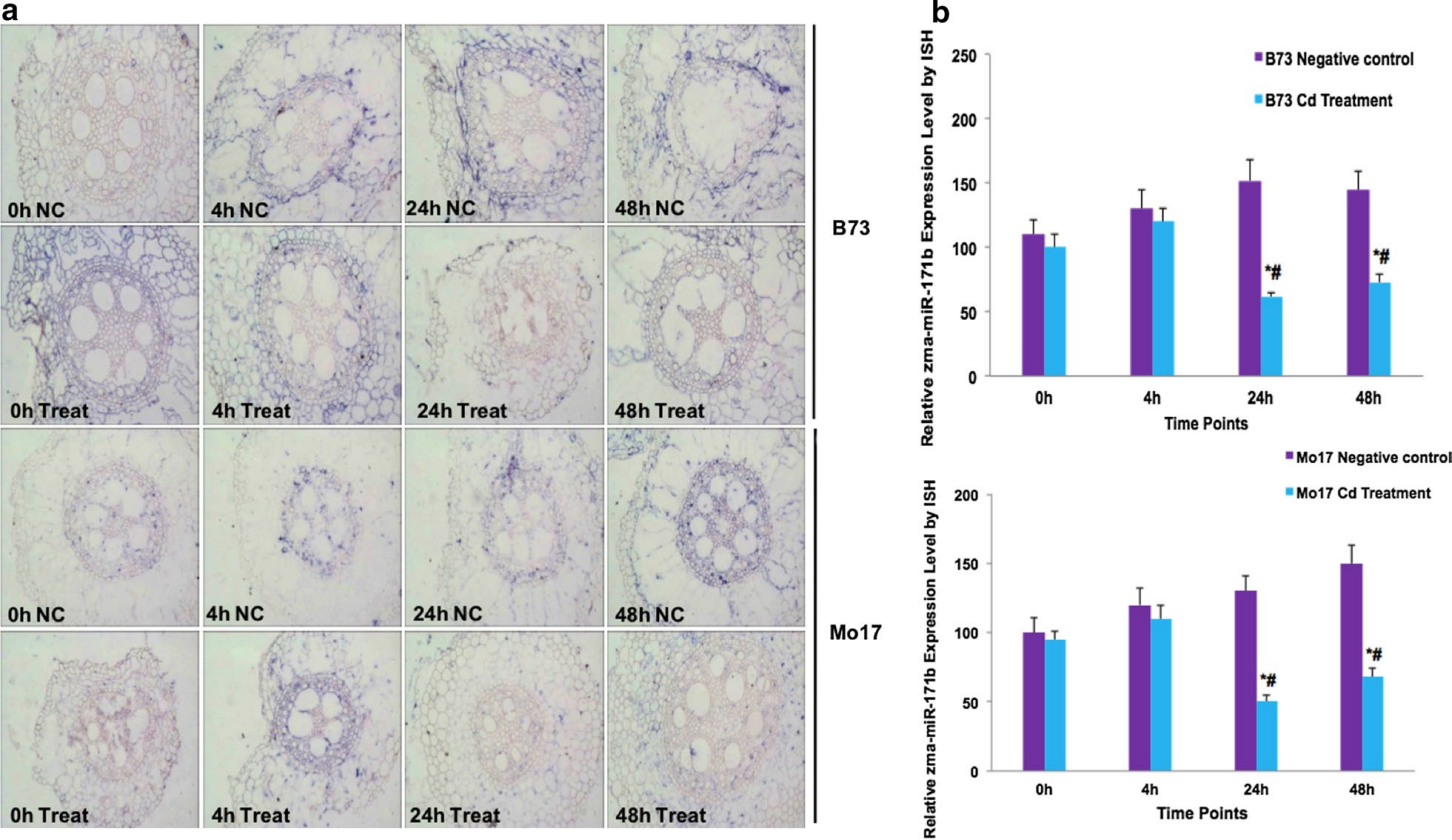
Change of relative quantity of Zma-miR171b in B73 and Mo17 using ISH. The microscopic results were quantified as black-blue (ISH) staining as a positive signal in per unit area (n = 9). Error bars show the standard error calculated from three biological replicates. Shown are the means per section ± SEM. *p < 0.05 treatment time vs. 0 h; ^#^p < 0.05 treatment vs. negative control (NC)

Based on the results obtained from the qRT-PCR of candidate miRNAs and their respective targets genes, the expression of *Zma*-*miR171b* was selected and further analyzed using in situ hybridization (ISH). Black-blue positive hybrid signals of *Zma*-*miR171b* were detected throughout at 0, 4, 12, and 24 h of treatment in maize roots of B73 and Mo17. Expression of *Zma*-*miR171b* was down-regulated following Cd stress at different stages in B73 roots, compared to that in the control, and reached a minimum at 48 h; similar trends were observed in Mo17, but expression levels of *Zma*-*miR171b* were consistently higher in B73 than in Mo17 (Fig. 4). *Zma*-*miR171b* was down-regulated in both phenotypes, thus B73 likely accumulated GRAS gene products earlier in response to Cd stress than Mo17 (Figs. 2e, 3e).

### Gene ontology annotation and analysis of target genes

Gene ontology (GO) analyses were performed to gain understanding of the function of target genes. Target genes that were specifically enriched in the regulation of biological processes showed transcription regulation activity and binding activity; however, those target genes were decreased in metabolism processes, and their catalytic activities were also reduced (Additional file 2: Figure S2).

## Discussion

miRNAs are one of the major types of endogenous, small, non-protein-coding single-stranded RNA, which act as regulators of plant growth and development at the translation and post-transcription levels by silencing gene expression [12, 13]. Previous studies demonstrated that miRNAs acts as regulators in plants exposed to abiotic stressors [14]. Moreover, many miRNAs were found to be important for plant responses to heavy metal stress using small RNA profiling [15, 16]. Recent studies identified numerous heavy metals such as Hg, Al, As, and Cd to regulate miRNAs in plants [6, 8–11]. However, we found that heavy metals regulated miRNAs varied from different plants and metal species. As shown in Fig. 1, some miRNA families (*miR396*, *miR390*, *miR171*, *miR166*, *miR162*, and *miR159*) were down-regulated, whereas *miR395* and *miR393* were up-regulated by most heavy metals. The expression of *miR396*, *miR390*, and *miR159* was consistently down-regulated, and *miR393* was up-regulated under exposure to four heavy metals. Ding et al. demonstrated that *miR390*, *miR171*, *miR168*, *miR166*, and *miR156* responded to Cd stress in rice [17], and Zhou et al. found that expression of *miR408*, *miR398*, *miR397*, and *miR396* was associated with Cd exposure in *Brassica* [5]. In addition, *miR393* and *miR171* were found to be affected by Cd stress through negatively regulating their target genes in *Oryza sativa* [18], *Medicago truncatula* [5], and *B. napus* [19]. In the current study, we demonstrated that *Zma*-*miR156b*, *Zma*-*miR166d*, *Zma*-*miR171b*, and *Zma*-*miR393b* were affected by Cd exposure in maize roots, using qRT-PCR-based analyses.

Recently, identification of target genes of metal-regulated miRNAs showed that most of these miRNAs encoded key regulatory proteins or TFs that were associated with diverse metabolic pathways [20–22]. In our study, several candidate miRNAs such as *Zma*-*miR156b*, *Zma*-*miR166d*, *Zma*-*miR167f*, *Zma*-*miR171b*, and *Zma*-*miR393b* were found to target the genes of TF families (SBP-box, bZIP, ARFs, GRAS), and F-box-like protein, most of which have been confirmed to play vital roles in the response to Cd stress (Additional file 1: Table S5, Fig. 4). Previous studies confirmed that SBP-box transcription factors were involved in a broad range of developmental and stress response processes targeted by *miR156* in *Arabidopsis* [23, 24]. In addition, the *SBP*-*box* gene family was characterized as metal-containing transcription factors regulating copper homeostasis in *Arabidopsis* [25]. In the present study, two miRNAs belonged to the *miRNA156* family (*Zma*-*miR156b* and *Zma*-*miR156k*) targeting *SBP*-*box* transcription factors. It may be inferred that *miR156* may be an important regulator in Cd^2+^ homeostasis in maize roots by targeting *SBP*-*box* transcription factors. Moreover, for the *bZIP* family, *bZIP62*, *ThbZIP1*, and *BjCdR15* were reported to be associated with Cd stress. Of those, the gene encoding bZIP62 was induced by Cd exposure in soybean roots, whereas *ThbZIP1* showed increased expression in *Tamarix hispida* [26, 27]. In the current study, we found that *bZIP* transcription factors were regulated by *Zma*-*miRNA166d*. We suggest that *Zma*-*miR166* acted in Cd^2+^ homeostasis in maize roots by targeting *bZIP transcription* factors. Furthermore, previous studies showed that some miRNAs responded to heavy metal stress through regulating phytohormone biosynthesis and signaling responses. For instance, As stress in *B. juncea* led to an increased *miR167* abundance [11]. The auxin response factors *ARF6* and *ARF8* targeted by *miR167* were found positively correlated with adventitious rooting and development of anthers and ovules via auxin homeostasis in *Arabidopsis* [28]. Moreover, ARF proteins may activate or repress the transcription of primary auxin response genes through binding to auxin-responsive elements [29]. F-box proteins, including transport inhibitor response 1 (TIR1) and auxin-signaling F-box proteins (AFB1, AFB2, and AFB3) can perceive auxins in plants [30–33]. In addition, *TIR1* encoded an E3 ubiquitin ligase containing F-box subunit were targeted by *miR393* [34] which interacts with Aux/IAA proteins as an auxin receptor, thereby resulting in Aux/IAA ubiquitination and subsequent degradation in auxin signal pathway [35]. In our study, we demonstrated that *Zma*-*miR393b* and *Zma*-*miR167f* targeted auxin signaling F-box proteins and that ARFs transcription factors responded to the Cd^2+^ treatment.

## Conclusion

In conclusion, this is the first study to characterize Cd-stress induced miRNAs and their target genes in maize roots. Six previously known miRNAs and their respective target genes were found to respond to Cd stress. The expression patterns of these differentially regulated miRNAs and association targets were shown to be affected by Cd stress in the inbred maize lines B73 and Mo17. Most of the target transcripts belonged to transcription factors that were predicted to be functionally involved in different pathways that respond to Cd stress. These results provide new insights for the functional characterization of miRNAs and their targets in plant responses to heavy metal stress.

## Methods

### Plant material and experimental design

The inbred maize line B73 was previously reported to be more tolerant to heavy metal stress than Mo17 [36], particularly regarding Cd stress. In this study, seeds collected from Mo17 and B73 were washed using distilled water and germinated at 26 °C in the dark for 3 days. Germinated seeds were grown in a growth chamber under the following conditions: a photoperiod of 14 h light/10 h dark and relative humidity of 70% at 26 °C. After 20 days, the seedlings were transplanted to a plastic container with a modified half-strength Hoagland nutrient medium (see Additional file 1: Table S1). Subsequently, the plants were randomly assigned to a Cd stress treatment (200 mg/L CdCl_2_·2.5 H_2_O) and a control (Cd-free).

### Enzymatic activity assays of POD and SOD

Maize seedlings of the treatment group and the control were collected after exposed to 200 mg/L CdCl_2_·2.5H_2_O for 0, 4, 8, 12, 24, 48, 72, or 96 h. POD and SOD activities were determined according to the methods described by Beyer [37] and Kim [38], respectively. All measurements were performed three times using three biological replicates.

### RNA isolation from maize roots

Based on the results of the POD and SOD enzymatic activity assays, total RNA was isolated from Cd-free and Cd-exposed roots of B73 and Mo17 after 0, 4, 8, 12, 24, and 48 h, using Trizol reagent (Life, US) following the manufacturer’s instructions. Small RNA was isolated from each sample using the mirVana™miRNA Isolation Kit (Ambion, US) according to the manufacturer’s instructions.

### Validation of candidate miRNAs using real-time PCR

Total miRNA was isolated from B73 and Mo17 using a Universal Plant microRNA Extraction Kit (Spin-column, BioTeke, China). Reverse transcription reactions of miRNAs were performed, and PCRs were performed in triplicate, according to Jian et al. [39]. Mitochondrial 5S RNA was used as a control, and specific primers were for real-time PCR (Additional file 1: Table S2). The absolute amount of each miRNA was calculated according to a standard curve using the 2^−∆∆CT^ method [40].

### ISH assays for *Zma*-*miR171b*

For ISH, miRCURY 5′-DIG and 3′-DIG-labeled locked nucleic acid (LNA)-miRNAs were utilized (Zma-miR171b) as detection probes. The sequence of the probe Zma-miR171b was 5′-DIG-UUGAGCCGUGCCAAUAUCAC-DIG-3′. Sections of 30 µm thickness were performed using LNA probes, and all steps were carried out as described by Urbanek et al. [41].

### Target gene prediction of candidate miRNAs in the Cd^2+^ treatment

To predict the accuracy of target genes, the online tools WMD3 (http://wmd3.weigelworld.org/cgi-bin/webapp.cgi?) and psRNA (http://plantgrn.noble.org/psRNATarget/) were used with default settings [42, 43].

### qRT-PCR confirmation of target genes

Specific qRT-PCR primers were designed for target genes using the software Primer Premier (version 5.0); the primers are shown in Additional file 1: Table S3. The amplification programs were performed according to the standard protocol of the ABI7500 system and were conducted in triplicate, according to the method of Jian et al. [39]. The cycle threshold (Ct) of each tested gene were averaged for triplicate reactions, and the values were normalized according to the Ct of the control products of the *Actin1* gene. Statistical analysis was performed using the 2^−∆∆CT^ method.

### GO annotation of putative target genes responsive to Cd stress

To annotate target genes we used singular enrichment analysis by examining the AgriGO database [44]. Using a customized or pre-calculated background, enriched GO terms were removed after statistical testing. GO annotation comprised biological processes, cellular components, and component functions of the putative sequences.

## Additional files

**Additional file 1: Figure S1.** Effect of Cd stress on SOD and POD enzyme activities in B73 and Mo17. The graphs (A) and (B) depicted the average change of superoxide dismutase (SOD) activities and contents of peroxidase (POD) in maize inbred line B73 and Mo17 leaf and roots respectively. The data are presented as the mean number per section ± SEM. *p<0.05 experimental vs. 0 h; #p<0.05 root vs. leaf. **Figure S2.** GO annotation of target genes of the candidate miRNAs. The Y -axis is the percentage of targeted genes mapped by the term, and represents the abundance of the GO term. The X -axis is the definition of GO terms.

**Additional file 2: Table S1.** The detailed components of the nutrient solution used in this study. **Table S2.** List of primers for qRT-PCR analysis of candidate miRNAs responsive to Cd stress. **Table S3.** List of primers for qRT-PCR analysis of targets related to those candidate miRNAs under Cd treatment. **Table S4.** Conserved analysis of candidate miRNAs responsive to heavy metal in different species. **Table S5.** Conserved candidate microRNAs related to Cd stress and their targets identified in maize.

## Abbreviations

Cd: cadmium
Al: aluminum
As: arsenic
Hg: mercury
SOD: superoxide dismutase
POD: peroxidase
miRNA: micro RNA
qRT-PCR: quantitative real-time PCR
ISH: in situ hybridization
SEA: singular enrichment analysis
GO: gene ontology
TF: transcription factor
ARF: auxin response factor
