# Supplementary material for: Characterization of cadmium-responsive MicroRNAs and their target genes in maize (Zea mays) roots
Source: BMC Mol Biol. 2019 May 2;20:14. doi: 10.1186/s12867-019-0131-1 (PMC6498490; doi:10.1186/s12867-019-0131-1)
Supplement: Supplementary file 2 — Additional file 2: Table S1. The detailed components of the nutrient solution used in this study. Table S2. List of primers for qRT-PCR analysis of candidate miRNAs responsive to Cd stress. Table S3. List of primers for qRT-PCR analysis of targets related to those candidate miRNAs under Cd treatment. Table S4. Conserved analysis of candidate miRNAs responsive to heavy metal in different species. Table S5. Conserved candidate microRNAs related to Cd stress and their targets identified in maize. [file 12867_2019_131_MOESM2_ESM.docx]

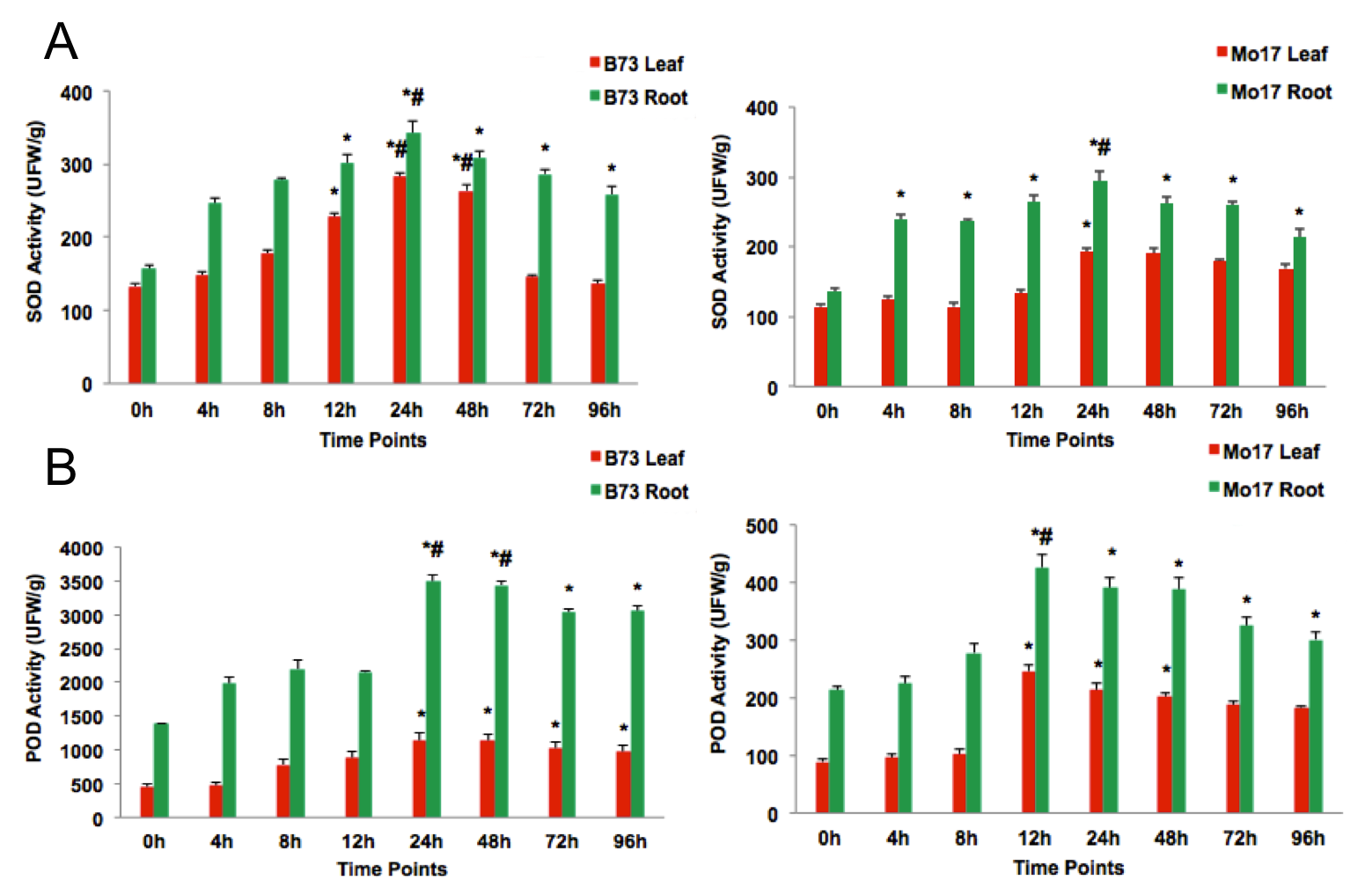


**Figure S1** Effect of Cd stress on SOD and POD enzyme activities in B73 and Mo17.

The graphs (A) and (B) depicted the average change of superoxide dismutase (SOD) activities and contents of peroxidase (POD) in maize inbred line B73 and Mo17 leaf and roots respectively. The data are presented as the mean number per section ± SEM. *p<0.05 experimental vs. 0 h; #p<0.05 root vs. leaf.


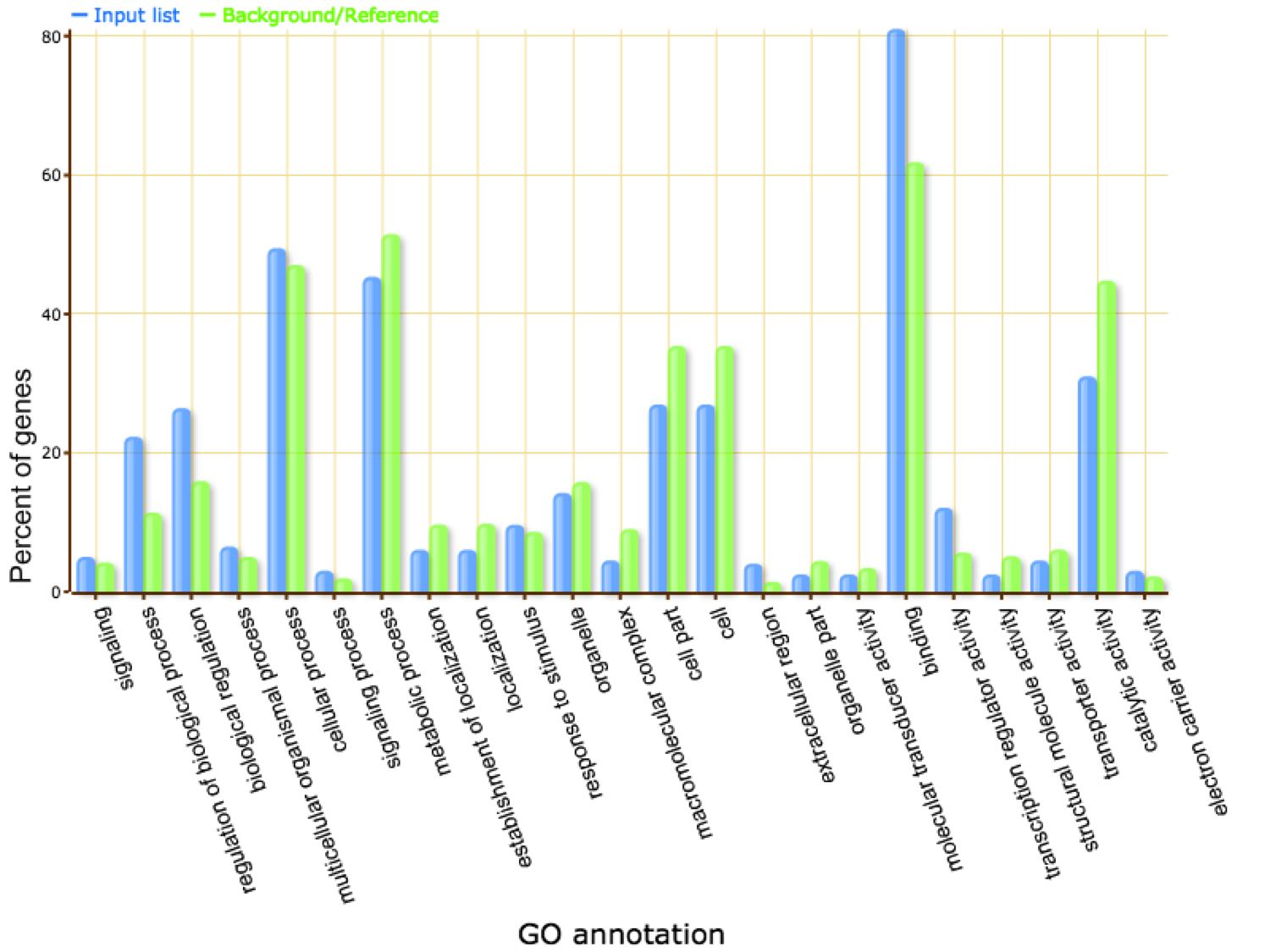


**Figure S2** GO annotation of target genes of the candidate miRNAs. The Y -axis is the percentage of targeted genes mapped by the term, and represents the abundance of the GO term. The X -axis is the definition of GO terms
